# Identification of Anaphase Promoting Complex Substrates in *S. cerevisiae*

**DOI:** 10.1371/journal.pone.0045895

**Published:** 2012-09-26

**Authors:** Denis Ostapenko, Janet L. Burton, Mark J. Solomon

**Affiliations:** Department of Molecular Biophysics and Biochemistry, Yale University, New Haven, Connecticut, United States of America; Texas A&M University, United States of America

## Abstract

The Anaphase-Promoting Complex/Cyclosome (APC/C) is an essential ubiquitin ligase that targets numerous proteins for proteasome-mediated degradation in mitosis and G1. To gain further insight into cellular pathways controlled by APC/C^Cdh1^, we developed two complementary approaches to identify additional APC/C^Cdh1^ substrates in budding yeast. First, we analyzed the stabilities of proteins that were expressed at the same time in the cell cycle as known APC/C substrates. Second, we screened for proteins capable of interacting with the Cdh1 substrate-binding protein in a yeast two-hybrid system. Here we characterize five potential APC/C substrates identified using these approaches: the transcription factors Tos4 and Pdr3; the mRNA processing factor Fir1; the spindle checkpoint protein kinase Mps1; and a protein of unknown function, Ybr138C. Analysis of the degradation motifs within these proteins revealed that the carboxyl-terminal KEN box and D-boxes of Tos4 are important for its interaction with Cdh1, whereas the N-terminal domain of Ybr138C is required for its instability. Functionally, we found that a stabilized form of Mps1 delayed cell division upon mild spindle disruption, and that elevated levels of Ybr138C reduced cell fitness. Interestingly, both Tos4 and Pdr3 have been implicated in the DNA damage response, whereas Mps1 regulates the spindle assembly checkpoint. Thus, the APC/C^Cdh1^-mediated degradation of these proteins may help to coordinate re-entry into the cell cycle following environmental stresses.

## Introduction

The orderly progression of cell cycle events requires the coordinated synthesis and degradation of multiple regulatory proteins. Degradation of these proteins occurs via the ubiquitin-dependent proteasome pathway, which entails sequential enzymatic reactions carried out by an E1 (ubiquitin activating enzyme), an E2 (ubiquitin conjugating enzyme), and an E3 (ubiquitin ligase). The resulting formation of poly-ubiquitin chains targets proteins for degradation by the 26S proteasome [1]. Two major classes of RING-type E3s that play critical roles targeting predominantly non-overlapping sets of proteins for degradation during cell cycle progression are the Anaphase-Promoting Complex/Cyclosome (APC/C) and the SCF complex [2]–[4].

In vegetative cells, the APC/C is a large complex composed of 13 distinct core proteins plus a WD40-repeat-containing activator, either Cdc20 or Cdh1, which recognize modular degradation motifs in substrate proteins. The most common motifs are the Destruction Box (D-box; RxxLxxxxN) and the KEN Box [5]–[7]. Mutations within these motifs eliminate recognition by Cdc20 and Cdh1, resulting in protein stabilization.

The activity of the APC/C is itself tightly regulated. Cdc20 is degraded during G1 in an APC/C^Cdh1^-dependent manner and is inhibited during mitosis by the spindle assembly checkpoint, which ensures that all chromosomes are properly attached to the mitotic spindle before the onset of anaphase [8]–[10]. Cdh1 is inhibited by phosphorylation by cyclin-dependent kinases and polo-like kinases, and by the binding of pseudosubstrate inhibitor proteins [11]–[15]. These regulatory processes limit Cdc20 activity primarily to mitosis and Cdh1 activity to the end of mitosis and G1. The ubiquitination of some APC/C substrates is further regulated by protein localization, phosphorylation, or interaction with other proteins [16], [17]. Thus, multiple factors contribute to determining when and how quickly individual proteins are degraded. The coordinated degradation of APC/C substrates ensures that cell cycle transitions are unidirectional.

APC/C substrates play important and diverse roles in the cell cycle. Cdc20 is an essential protein and the degradation of two of its substrates, Pds1/securin and the cyclin Clb5, are essential for cell viability in yeast [18], [19]. Another important APC/C^Cdc20^ substrate is Mps1, an essential protein kinase involved in spindle pole body (SPB; functional equivalent of the centrosome) duplication and the spindle assembly checkpoint [20], [21]. In contrast to Cdc20, yeast cells lacking Cdh1 are viable, although they grow more slowly than wild-type cells and exhibit various morphological defects [22]. Efficient disassembly of the mitotic spindle requires Cdh1-mediated degradation of the kinesins Cin8 and Kip1 and of two microtubule-associated proteins, Ase1 and Fin1 [23]–[26]. Cdh1 also participates in transcriptional activation during G1 by mediating the degradation of two transcriptional repressors, Nrm1 and Yhp1 [27].

Since stabilization of individual APC/C^Cdh1^ substrates has only modest effects, it is likely that the combined and coordinated degradation of multiple APC/C^Cdh1^ substrates is essential for normal cell growth. To further understand cell cycle pathways regulated by APC/C, we developed complementary systematic approaches to identify additional Cdh1 substrates. After validating these proteins, we explored whether stabilization of two of them might affect cell proliferation. Although the detailed functions of these proteins are largely unknown, two of them are implicated in the response to DNA damage and one is involved in the spindle assembly checkpoint. Thus, it appears that by targeting a few regulatory proteins, APC/C^Cdh1^ may help to coordinate recovery from checkpoint arrests with re-entry into the cell cycle.

## Materials and Methods

### Yeast strains and plasmids

Yeast strains were derivatives of W303a (*ade2-1 trp1-1 leu2-3*,*112 his3-11*,*15 ura3-1*) [28]. The *cdh1Δ* strain and the temperature-sensitive *cdc23-1*, *cdc15-2*, and *cdc28-13 cdh1Δ* strains were described previously [14], [29]. The *MET-CDC20* strain was a generous gift of Angelika Amon (MIT, Cambridge, MA) [30]. *cdh1-m11* was provided by Wolfgang Seufert (University of Stuttgart, Stuttgart, Germany) [15]. The yeast two-hybrid strains pJ69-4a and pJ69-4α were provided by Stan Fields (University of Washington, Seattle, WA) [31]. Construction of *ybr138CΔ* (W303a *ybr138C::natMX4*) strains was accomplished by a PCR-based method [32]. Gene disruptions were verified by PCR using a primer downstream of the deleted gene and a primer internal to *natMX4*.

The *CLB2-TAP*, *TOS4-TAP* and *PDR3-TAP* strains were isolated from a TAP library [33] and cell extracts from these strains were used in Cdh1-binding assays (see below). *FIR1-TAP*, *MPS1-TAP*, and *YBR138C-TAP* were amplified from the TAP library [33] and cloned into YCplac22-GAL [34]. The resulting plasmids were used as templates to introduce mutations within putative regulatory motifs. For *MPS1*, the following sites were altered: ^267^RELL->^267^AELA, ^319^RRAL->^319^ARAA, and ^356^REVL->^356^AEVA (*mps1-3mdb*). For *YBR138C*, we generated an N-terminal deletion (*ybr138C-ΔN80*) and three point mutations: ^177^RPRL->^177^APRA, ^462^RLQL->^462^ALQA, and ^495^RRKL->^495^ARKA (*ybr138C-3mdb*).

*TOS4-HA-*YIplac211 and *PDR3-HA-*YIplac211 were made by insertion of the corresponding gene coding sequence into YIplac211 [34] encoding three copies of the HA-epitope tag. The resulting plasmids were integrated into the yeast genome at the endogenous gene loci to tag the chromosomal copies of these genes. *GAL-TOS4-HA*-YIplac204 and *GAL-PDR3-HA*-YIplac204 were made similarly except that the genes were placed in a *GAL-*YIplac204 plasmid containing three copies of the HA-epitope tag. These plasmids were integrated into the *TRP1* locus.

All mutations (including those described below) were verified by DNA sequencing of the entire coding regions by the Keck Facility (Yale University, New Haven). Primer sequences and further details of the plasmids are available upon request.

### Cell growth and arrest

Cultures were grown in YPD and in complete minimal (CM) media as described [35]. Cells of *bar1Δ* strains were arrested in G1 phase with 100 ng/ml alpha-factor or in M phase with 20 µg/ml benomyl for 2 h at 30°C. Inactivation of APC/C^Cdh1^ activity was achieved by incubation of *cdc23-1* cells at 37°C for 1 hour following cell cycle arrest. *MET-CDC20*-expressing cells were synchronized by incubation with 5 mM methionine for 2 h at 30°C; cells were washed by filtration (Corning Filter System) and released into pre-warmed methionine-free medium. *cdc15-2* cells were synchronized by arrest in anaphase by incubation at 37°C for 3 hours followed by release at 23°C. For analyses of protein stability, cells were grown in YP-raffinose to mid-exponential phase (OD_600_∼0.4). Galactose was added to 2% for 50 min at 30°C (or 60 minutes for *GAL-TOS4-HA* and *GAL-PDR3-HA* strains), followed by addition of cycloheximide (500 µg/ml, MP Biomedicals) and 2% dextrose as described [14]. For protein stability assays of endogenous Tos4 and Pdr3 in asynchronous cells, either wild-type (WT) or *cdh1Δ* strains expressing endogenous levels of Tos4-HA and Pdr3-HA were grown to OD_600_∼0.6 at which point cycloheximide (500 µg/ml, MP Biomedicals) was added for the indicated times.

For co-culture experiments, two different protocols were used. In the first protocol, *MPS1* and *mps1-3mdb* mutant strains were genetically marked (with *TRP1* and *HIS3*) and grown together in YPD supplemented with 50 µg/ml of tryptophan and histidine at 30°C in the presence and absence of 6 µg/ml nocodazole. The cultures were diluted 1000-fold into fresh medium each day and tested for the presence of Trp and His auxotrophs by plating on selective media. In the second protocol, 2x*YBR138C* (where an extra copy of *YBR138C* was inserted into the *URA3* locus) and wild-type (W303) cells were marked (with *TRP1* and *LEU2*) and grown together in YPD supplemented with 50 µg/ml of tryptophan and leucine. The cultures were diluted 1000-fold into fresh medium each day and tested for the presence of *TRP1* and *LEU2* auxotrophs by plating on selective media. Cultures were grown to an OD_600_ between 1.0 and 2.0 prior to daily dilution. In both protocols, the markers were swapped, the experiment repeated, and the results averaged.

The relative fitness of a mutant strain was defined as the fraction of a cell cycle that the strain underwent in the time the corresponding wild-type strain underwent one cell cycle. This value can be obtained from the formula: f = 1+ln(R)/(a^.^ln(2)), where f is the relative fitness of the mutant strain, R is the ratio of the number of mutant cells to the number of wild-type cells late in the growth of the co-culture (normalized to a ratio of 1 when the culture was started), and “a” is the number of generations undergone by the culture at the time of analysis. (More precisely, “a” should be the number of generations undergone by the wild-type cells, but the number of generations undergone by the culture is easier to determine and, as here, when the growth rates are similar, differs from the desired number by less than the error in the measurements.) This calculation is more accurate than the one we used previously [27]. Using this formula, the reductions in fitness for the Nrm1-mdb and Yhp1-mkb/mdb strains [27] are approximately 12.4% and 7.1%, respectively. All fitness calculations were verified by simulation using an Excel spreadsheet.

### Yeast extracts and immunoblotting

Cell extracts were prepared by shaking cell suspensions with glass beads as described [14]. Proteins were separated by SDS-PAGE and transferred to an Immobilon-P membrane (Millipore). (Low-abundance TAP-tagged proteins were precipitated with IgG-Sepharose (GE Healthcare) prior to analysis.) TAP-tagged proteins were detected by probing the membranes with peroxidase-anti-peroxidase (PAP, 1.3 µg/ml, Sigma) antibodies followed by visualization by chemiluminescence (SuperSignal, Pierce). Tos4-HA and Pdr3-HA were detected using anti-HA antibodies (12CA5, 0.5 µg/ml, Covance). Cdc28 was detected with anti-PSTAIR antibodies [36].

### Cdh1-binding Assays

Detection of binding to 6xHis-Cdh1 beads was performed essentially as described [5] except that cell extracts harboring endogenous levels of the TAP-tagged proteins were incubated with the Cdh1-beads and binding was visualized using PAP antibodies (1.3 µg/ml).

### Yeast two-hybrid screening

*CDH1-ΔN200*-pAS2 (*CDH1*), *cdh1-ΔN200-D12*-pAS2 (*cdh1-D12*), *HSL1^667–872^*-pACTII (*HSL1*) and *ACM1*-pACTII plasmids were described previously [37]. The *TOS4*-pACT plasmid isolated in the yeast two-hybrid screen contained the entire open reading frame of *TOS4*. The indicated regions of *TOS4* (*N267*, *C222*, *ΔC125* and *C222Δ125*) were cloned into the pACTII plasmid such that the corresponding coding regions of *TOS4* were in frame with the *GAL4*-activation domain in pACTII. The *TOS4-C222*-pACTII construct was mutated by inverse PCR to introduce the following mutations: ^365^KEN->^365^AAA, ^414^RDEL->^414^ADEA, ^418^RSIL->^418^ASIA, ^458^RRNL->^458^ARNA, and ^469^RTGL->^469^ATGA (*C222-mut.5*).

The pJ69-4a strains containing the indicated bait plasmids were mated with pJ69-4α strains harboring the indicated activation domain plasmids. Diploids were selected for on medium lacking leucine and tryptophan. For detection of the two-hybrid interaction, isolated diploids were struck onto plates lacking leucine, tryptophan, histidine and adenine. Screening of the yeast two-hybrid library was performed essentially as described [29], [37] using pJ69-4a cells expressing the *CDH1-ΔN200* bait plasmid. *GAL4* activation domain library plasmids were transformed into the *CDH1* bait strain and directly selected for the two-hybrid interaction by plating onto medium lacking leucine, tryptophan, histidine and adenine. Strains that were able to grow on selective medium were induced to lose the *CDH1* bait plasmid by growth in non-selective medium and then mated with pJ69-4α strains containing the *SNF1*, *CDH1* or *cdh1-D12* bait plasmids. Activation domain plasmids that interacted with wild-type *CDH1* but not with *cdh1-D12* strains were isolated and sequenced.

### Ubiquitination Assays

Tos4, Tos4-N267, Tos4-C222 and Tos4-C222 mutant 5 were radiolabeled *in vitro* with ^35^S-methionine (Perkin Elmer) using the TNT T7 quick-coupled transcription-translation system (Promega) as previously described [14]. Ubiquitination assays were performed using purified Uba1 and APC/C from yeast, recombinant yeast Ubc4 produced and purified from *E. coli*, and yeast 6xHis-Cdh1 produced and purified from baculovirus-infected insect cells as previously described [14]. Ubiquitinated proteins were visualized by autoradiography.

## Results

### Identification of candidate APC/C substrates by transcriptional profiling

Even though essential APC/C targets have been described, it is likely that numerous ‘minor’ substrates remain to be identified. We set up systematic screens to identify additional proteins that are targeted by APC/C^Cdh1^ in *S. cerevisiae*. The first approach was based on an analysis of the transcriptional profiles of known and putative APC/C substrates, an approach we used previously to identify Nrm1, Yhp1 and Iqg1 as APC/C substrates [27], [38]. We previously reported that transcription of the genes encoding all known APC/C substrates are cell cycle regulated and could be divided into two clusters based on the timing of their expression [27]. The first cluster includes genes expressed in G1 that encode APC/C substrates such as Hsl1, Fin1 and Cin8, whereas the second cluster includes genes expressed in G2 phase, previously known as the *CLB2* cluster, encoding APC/C substrates such as the mitotic cyclins, Ase1, Cdc20 and Cdc5.

**Figure 1:**
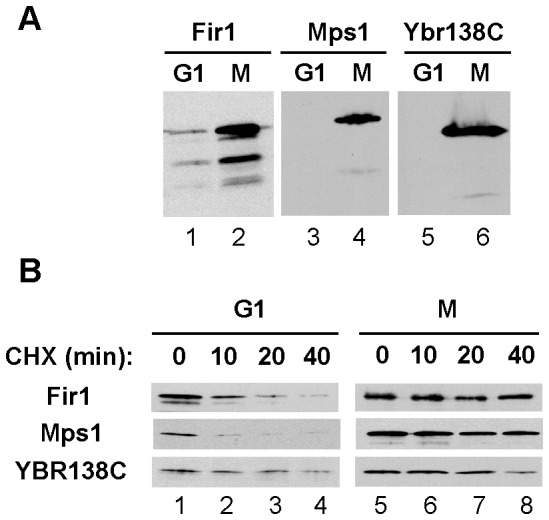
Identification of candidate APC/C substrates by protein expression profiling. (**A**) Analysis of protein levels in G1 and M. Cells expressing the indicated TAP-tag fusion proteins were arrested in G1 or M phase and the corresponding cell extracts were immunoblotted with PAP antibodies to detect TAP-tagged proteins. (**B**) Protein degradation in G1 and M. Cells were arrested in either G1 or M phase and treated with 500 µg/ml cycloheximide for the indicated times. Cell extracts were prepared and immunoblotted to examine TAP-tag protein levels as in (A).

This coherent transcriptional regulation suggested that novel APC/C substrates are likely to share the same transcriptional profiles. Therefore, we systematically analyzed yeast proteins that were co-expressed with known APC/C substrates within the G1 and G2 clusters, paying special attention to those that were also reported to have relatively short half-lives [39]. The abundance of 134 candidate proteins was examined at two stages of the cell cycle: in G1, when APC/C^Cdh1^ is highly active, and in spindle checkpoint-activated cells when all forms of the APC/C are inactive. Proteins that were less abundant in G1 cells than in M phase cells were considered further. Using this approach, we found that Fir1, Mps1, and Ybr138C were depleted from G1-arrested cells (Figure 1A). This change in protein abundance might result from differences in gene transcription, protein stability, or both. To distinguish among these possibilities, we examined the half-lives of candidate proteins in G1 and M phases. We found that the abundance of Fir1, Mps1 and Ybr138C declined in G1 cells following the inhibition of protein synthesis by the addition of cycloheximide (Figure 1B, lanes 1–4). In contrast, these proteins were relatively stable following cycloheximide addition to spindle checkpoint-arrested M phase cells (Figure 1B, lanes 5–8). These experiments suggested that APC/C^Cdh1^ might target Fir1, Mps1, and Ybr138C for proteasome-mediated degradation. Thus, these proteins were further characterized as described below. While this work was in progress, others found that Mps1 was an APC^Cdc20^ substrate [40].

### Fir1, Mps1, and Ybr138C are APC/C^Cdh1^ substrates

**Figure 2:**
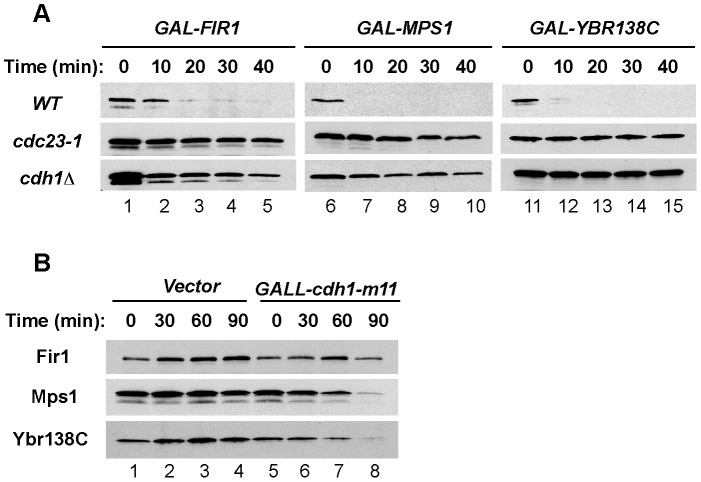
APC/C^Cdh1^-dependent degradation of Fir1, Mps1, and Ybr138C in G1. (**A**) Wild-type and *cdc23-1* cells were arrested in G1 with alpha factor and transferred to 37°C to inactivate Cdc23-1, a core subunit of the APC/C. *cdc28-13 cdh1Δ* cells were arrested in G1 by incubation at the non-permissive temperature (37°C). The expression of *FIR1*, *MPS1*, *and YBR138C* was induced from *GAL1p* followed by addition of 2% dextrose and 500 µg/ml cycloheximide to terminate protein synthesis. Samples were withdrawn at the indicated times and processed for immunoblotting with PAP antibodies. (**B**) Cells carrying an empty vector (lanes 1–4) or *GALLp-cdh1-m11* (lanes 5–8) were grown in the presence of raffinose and induced with 2% galactose for the indicated times. Cdh1-m11 lacks sites of inhibitory phosphorylation and is constitutively active. Samples were processed for immunoblotting to examine endogenous levels of Fir1, Mps1, and Ybr138C.

To determine directly if the APC/C promoted the degradation of Fir1, Mps1, and Ybr138C, we carried out half-life experiments in G1-arrested wild-type and APC/C mutant cells. Upon transient galactose-induced expression to approximately endogenous levels, we found that all three proteins were rapidly degraded in wild-type cells but were stable after temperature inactivation of the Cdc23 core subunit of the APC/C or in cells lacking Cdh1 (Figure 2A), indicating that APC/C^Cdh1^ was required for their degradation.

Additional evidence that APC/C^Cdh1^ was directly involved in the degradation of the identified proteins was obtained using a constitutively-active form of Cdh1, Cdh1-m11, which carries substitutions within eleven phosphoacceptor sites, rendering it refractory to Cdc28-mediated inhibition [15]. Expression of *cdh1-m11* promoted declines in the levels of Mps1 and Ybr138C (Figure 2B, lanes 5–8), comparable to the declines previously found for the known APC/C substrates Clb2 and Nrm1 [27].

### N-terminal sequences are required for Fir1 and Ybr138C degradation

**Figure 3:**
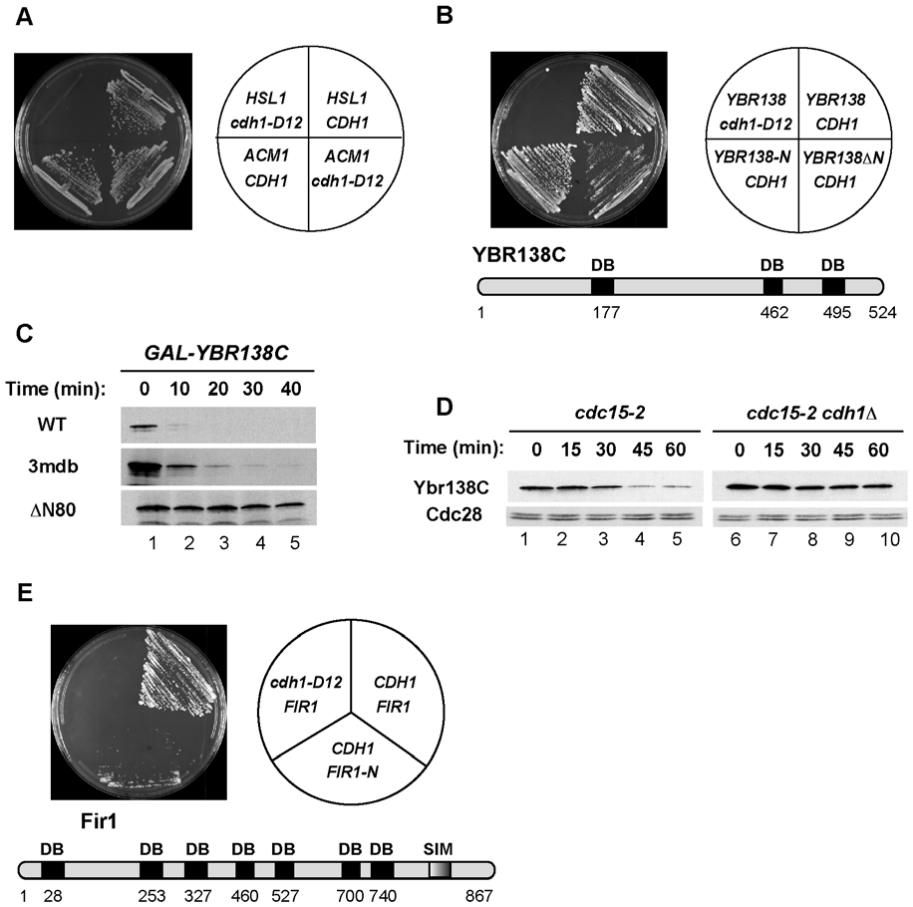
Degradation of Ybr138C and Fir1 and their interaction with Cdh1. (**A**)Two hybrid assay to detect Cdh1-substrate interactions. The bait in this assay was either wild-type Cdh1-ΔN200 or Cdh1-ΔN200-D12, which contains mutations within Cdh1's WD40 domain previously demonstrated to disrupt Cdh1's interaction with the substrate D-Box [37], [41]. The prey consisted of the APC/C substrate Hsl1 or the APC/C pseudosubstrate inhibitor Acm1 [37]. (**B**) Yeast two-hybrid interaction between Cdh1 and Ybr138C. Cells expressing Cdh1 or Cdh1-D12 and Ybr138C, Ybr138C-ΔN200, or Ybr138C-N270 were tested for growth on selective medium, which indicates interaction of the respective proteins. Bottom: Schematic representation of Ybr138C indicating positions of potential D-boxes (DB). (**C**) Degradation of Ybr138C requires its N-terminal region but not putative D-boxes. Cells expressing wild-type *YBR138C*, *YBR138C-3mdb*, and *YBR138C-ΔN80* from a *GAL1* promoter were arrested in G1 and induced with galactose. Samples were withdrawn at the indicated times after cycloheximide addition and processed for blotting with PAP antibodies as in Fig. 2A. (**D**) *cdc15-2* and *cdc15-2 cdh1Δ* cells carrying endogenous *YBR138C-TAP* were synchronized in mitosis by incubation at 37°C for 3 hours and released at 23°C at time zero. Samples were withdrawn at the indicated times and processed for immunoblotting. Cdc28 was used as a loading control. (**E**) Yeast two-hybrid interaction between Cdh1 and Fir1. As in (B) but cells carried Fir1 or Fir1-N200 instead of Ybr138C. Bottom: Schematic of Fir1 indicating its potential D-boxes (DB) and its SUMO-interacting motif (SIM).

We further probed candidate APC/C substrates by examining their interactions with Cdh1 using a yeast two-hybrid assay. These assays were performed using a form of Cdh1 lacking its first 200 amino acids (Cdh1-ΔN200, termed Cdh1 below for simplicity) since their presence causes activation in the two-hybrid system even in the absence of a prey plasmid. Using this assay we previously demonstrated that Cdh1 interacted with Hsl1, a well-characterized APC/C substrate, and with Acm1, a pseudosubstrate regulator of APC/C^Cdh1^ that contains a Destruction Box, a KEN box, and a third interaction sequence termed the A-motif [37]. Mutation of amino acid residues within the WD40 domain of Cdh1 (Cdh1-D12) that mediate D-box-binding disrupted the interaction of Cdh1 with its substrate, Hsl1, but not with Acm1, thus providing an additional tool for identifying Cdh1-binding proteins that are APC/C substrates [37], [41] (Figure 3A and see below). In addition, this system can be used to identify D-Boxes and KEN boxes within APC/C substrates that promote their interaction with Cdh1.

As with Hsl1, Ybr138C and Fir1 interacted strongly and specifically with Cdh1 but not with Cdh1-D12 (Figures 3B and 3E). We used these two-hybrid interactions in an initial attempt to identify potential degradation motifs within Ybr138C and Fir1. We found that deletion of the N-terminal 200 amino acids of Ybr138C reduced its interaction with Cdh1, whereas this N-terminal region alone interacted with Cdh1 (Figure 3B). Surprisingly, mutation of three putative D-boxes within Ybr138C, ^177^RPRL, ^462^RLQL, and ^495^RRKL individually and in combination, had little effect on the two-hybrid interaction with Cdh1 (data not shown), suggesting that Ybr138C binds Cdh1 through a different motif. Neither this triple mutant (Ybr138C-3xmdb) nor additional mutations within potential KEN- and D-box-like sequences (^293^EEN and ^9^RVL) had significant effects on the stability of Ybr138C in G1-arrested cells (Figure 3C and data not shown). In contrast, deletion of the N-terminal 80 amino acids stabilized Ybr138C (Figure 3C, bottom panel). Mutation of the three putative D-boxes conferred no additional stabilization (data not shown), indicating that the N-terminal sequence was responsible for Ybr138C degradation. These findings also validate the use of the yeast two-hybrid system to identify which region(s) of an APC/C substrate are involved in Cdh1-interaction and protein degradation.

We examined the cell cycle variations in Ybr138C levels in synchronized *cdc15-2* cells that were arrested in anaphase, released, and followed through the next cell cycle. In cells with wild-type *CDH1*, Ybr138C levels were high in mitosis but declined approximately 30 minutes after release from anaphase arrest, when cells entered the next G1 phase (Figure 3D, lanes 1–5). In contrast, Ybr138C levels remained high after release in *cdc15-2 cdh1*Δ cells (Figure 3D, lanes 6–10), indicating that APC/C^Cdh1^ was responsible for the degradation of Ybr138C in the *cdc15-2* cells.

As with Ybr138C, Fir1 contains numerous putative D-boxes whose individual mutation did not stabilize Fir1 in G1-arrested cells (data not shown). Using the yeast two-hybrid system we found that the N-terminal 200 amino acids of Fir1 were sufficient for interaction with Cdh1, whereas further deletions significantly reduced the interaction, suggesting that the Cdh1-binding sequences are located within the N-terminal portion of Fir1 (Figure 3E). We also analyzed the cell cycle fluctuations of Fir1 in cells synchronized in mitosis by Cdc20 depletion. As with Ybr138C, Fir1 levels were high in mitosis but declined in the next G1 phase, consistent with Fir1 being an APC substrate (data not shown).

### Tos4 and Pdr3 are potential APC/C^Cdh1^ substrates

**Figure 4:**
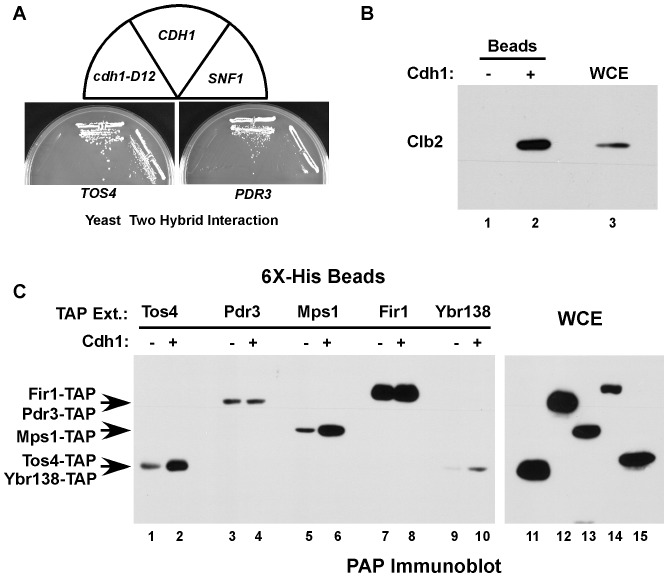
Cdh1 bead binding assay for TAP-tagged fusion proteins from whole cell extracts. (**A**) Tos4 and Pdr3 interact with wild-type Cdh1 but not with Cdh1-D12 in the yeast two-hybrid system. (**B**) A Cdh1-bead binding assay. An extract from cells expressing Clb2-TAP was incubated with the indicated beads and binding was detected using PAP antibodies. WCE, whole cell extract. (**C**) *Left panel*: Extracts of cells expressing the indicated TAP-tag fusion proteins were incubated with Cdh1 beads or control beads and the levels of bound TAP-tagged proteins were detected by blotting with PAP antibodies (lanes, 1–10). *Right panel*: 2% of the input material (whole cell extract, WCE) for the binding assays is shown (lanes 11–15).

Based on the above yeast two-hybrid interactions between Cdh1 and APC/C substrates (Figs. 3A, 3B, and 3E), we conducted a two-hybrid screen using Cdh1-ΔN200 (*CDH1*) as the bait protein to isolate additional potential APC/C^Cdh1^ substrates. Positive clones were induced to lose the Cdh1 bait protein and then mated to *SNF1*, *CDH1* or *cdh1-D12* bait strains to identify clones that interacted specifically with wild-type Cdh1 but not the cdh1-D12 mutant. Two of the strains passing these tests contained *TOS4* and *PDR3* activation domain plasmids (Figure 4A). *TOS4* (Target of Sbf 4) encodes a putative forkhead-like transcription factor. *TOS4* expression is periodic and is similar to the G1 cluster transcription profile of *HSL1*, *FIN1* and *CIN8* described above [42]. *PDR3* (Pleiotropic drug resistance 3) encodes a transcription factor that up-regulates the expression of the ABC-transporter protein Pdr5 involved in the pleiotropic drug resistance pathway [43].

Validation for these two-hybrid interactions can be obtained by examining binding between Cdh1 and its potential targets *in vitro* using recombinant Cdh1 bound to beads. For instance, a Clb2-TAP tag fusion protein in a cell extract bound specifically to Cdh1-containing beads (Figure 4B), similar to what we had previously found for Hsl1 [5]. Thus, candidate APC/C substrates found to interact with Cdh1 in the yeast two-hybrid system can be tested for Cdh1-binding in this biochemical assay using available yeast strains expressing the appropriate TAP-tag fusion protein. Furthermore, candidate substrates can be tested for ubiquitination *in vitro* using purified E1, E2 and APC/C^Cdh1^ components, as has been shown for Hsl1 and other substrates (data not shown and see below).

We tested the potential APC/C-substrates identified above by two-hybrid screening or transcriptional profiling for Cdh1-binding *in vitro*. Cell extracts expressing endogenous levels of each of the TAP-tag fusion proteins were incubated with control or Cdh1-containing Talon beads. Bound proteins were detected with PAP antibodies (Figure 4C, lanes 1–10). Tos4, Mps1 and Ybr138C all bound to Cdh1 beads, with only minimal binding to the control beads, providing additional evidence that they are APC/C substrates. It is currently unclear why Pdr3 and Fir1 bound equally well to both Cdh1 and control beads.

**Figure 5:**
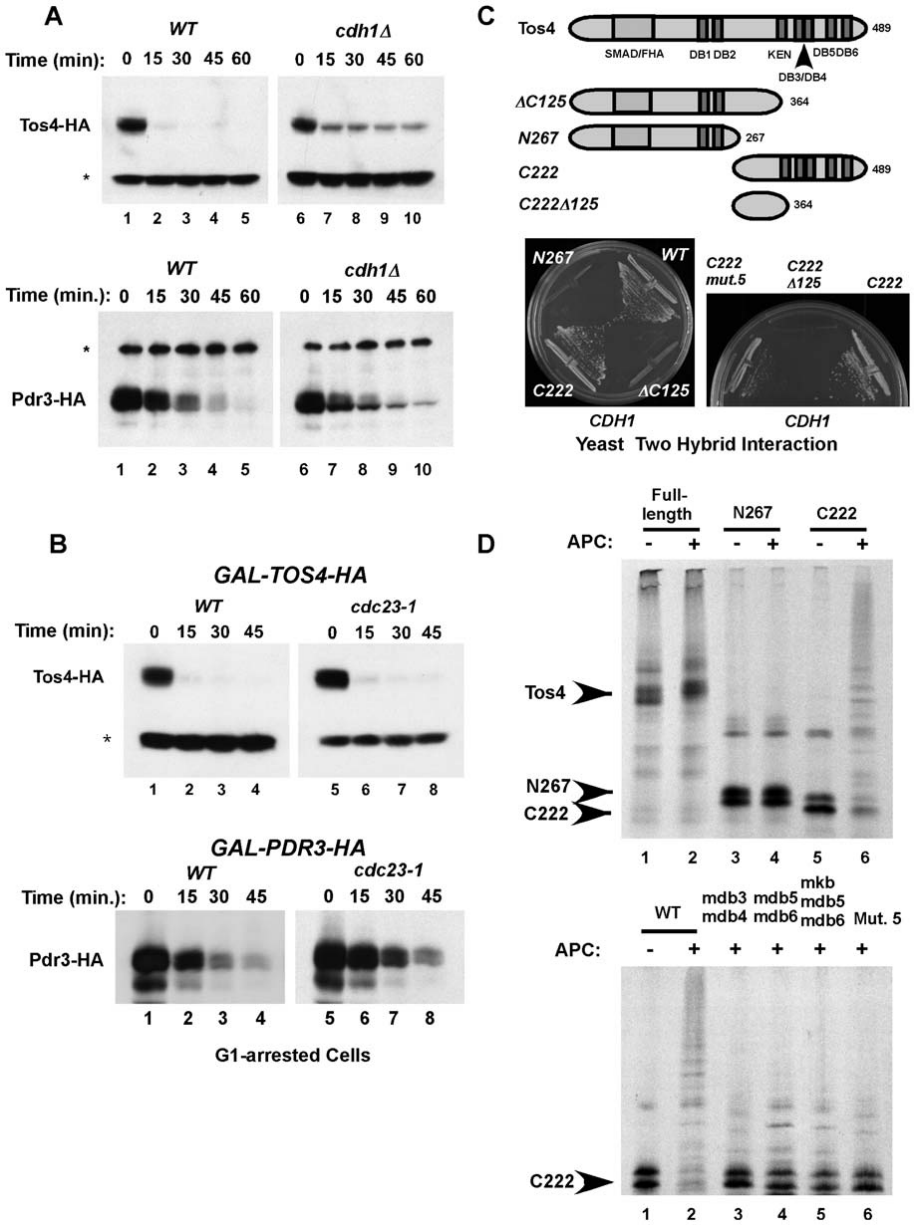
Tos4 and Pdr3 are potential APC/C^Cdh1^ substrates. (**A**) Tos4 and Pdr3 are partially stabilized in *cdh1Δ* cells. Asynchronously growing WT or *cdh1Δ* cells expressing Tos4-HA or Pdr3-HA were treated with cycloheximide for the indicated times. HA-fusion protein levels from the cell extracts were determined by immunoblotting with anti-HA antibodies. A non-specific cross-reactive protein (*) served as a loading control. (**B**) Overexpressed Pdr3-HA but not Tos4-HA was partially stabilized in an APC/C mutant strain. Cells were arrested in G1 and induced to express the indicated HA-tagged protein by galactose addition. Cells were shifted to 37°C to inactivate the APC/C in the *cdc23-1* mutant cells, and then treated with cycloheximide for the indicated times prior to extract preparation and protein detection by immunoblotting with anti-HA antibodies. (**C**) The carboxyl-terminal region (C222) of Tos4 contains multiple motifs that mediate interaction with Cdh1 in the yeast two-hybrid system. Top: Schematic of Tos4 constructs showing the locations of the putative KEN box and DB motifs. Tos4-mut. 5 contains mutations of the four C-terminal D-box and one KEN box motifs. (**D**) Tos4-C222 was efficiently ubiquitinated by APC/C^Cdh1^ *in vitro*. The N267 and C222 forms of Tos4 are shown in (C), as are the sites of the various DB mutations. Locations of the putative Tos4 degrons are as follows: DB1, 232; DB2, 237; KEN, 365; DB3, 414; DB4, 418; DB5, 458; DB6, 469.

We examined the stability of Tos4 and Pdr3 after cycloheximide addition to asynchronously growing cell cultures. Endogenously expressed Tos4-HA and Pdr3-HA were both unstable and each was partially stabilized in cells lacking Cdh1 (*cdh1Δ* cells; Figure 5A, lanes 6–10). We also examined the stability of overexpressed Tos4-HA and Pdr3-HA in G1-arrested WT and APC/C mutant (*cdc23-1*) cells (Figure 5B). Pdr3-HA was partially stabilized in *cdc23-1* cells whereas Tos4-HA stability was not affected by APC/C inhibition under these conditions. Thus, both Tos4 and Pdr3 are unstable proteins whose degradation is regulated by both APC/C^Cdh1^ and at least one additional ubiquitin ligase.

### The Tos4 carboxyl-terminus interacts with Cdh1 and is ubiquitinated *in vitro*

Tos4 contains six putative D-boxes and one KEN box that could potentially mediate its interaction with Cdh1 and promote its degradation. To determine which regions of Tos4 bind Cdh1, we tested different portions of Tos4 (Figure 5C schematics) in the yeast two-hybrid assay for interaction with the *CDH1* bait plasmid. Only full-length Tos4 and its last 222 amino acids (C222) interacted with Cdh1 in this assay whereas the amino-terminus of Tos4 (N267) or Tos4 lacking its final 125 amino acids (ΔC125) could not (Figure 5C). Interestingly, the last 125 amino acids of Tos4 contained 4 out of its 6 potential D-boxes (DB) as well as its KEN box (Figure 5C). Mutation of all five motifs within C222 (C222-mut. 5) greatly reduced the two-hybrid interaction with Cdh1 (Figure 5C). The residual binding suggests that an additional unknown motif within Tos4-C222 also interacts with Cdh1, albeit weakly.

We next determined if Tos4 could be ubiquitinated by APC/C^Cdh1^ *in vitro*. Tos4-C222 was efficiently ubiquitinated, although full-length Tos4 and Tos4-N267 were not (Figure 5D, top panel). Furthermore, mutation of the D-boxes and the KEN box within Tos4-C222 virtually eliminated ubiquitination (Fig. 5D, bottom panel). The strongest effect was shown by mutations of DB3 and DB4, suggesting that these motifs mediate binding to Cdh1 and Tos4 turnover.

### Elevated expression of Ybr138C reduces cell fitness

**Figure 6:**
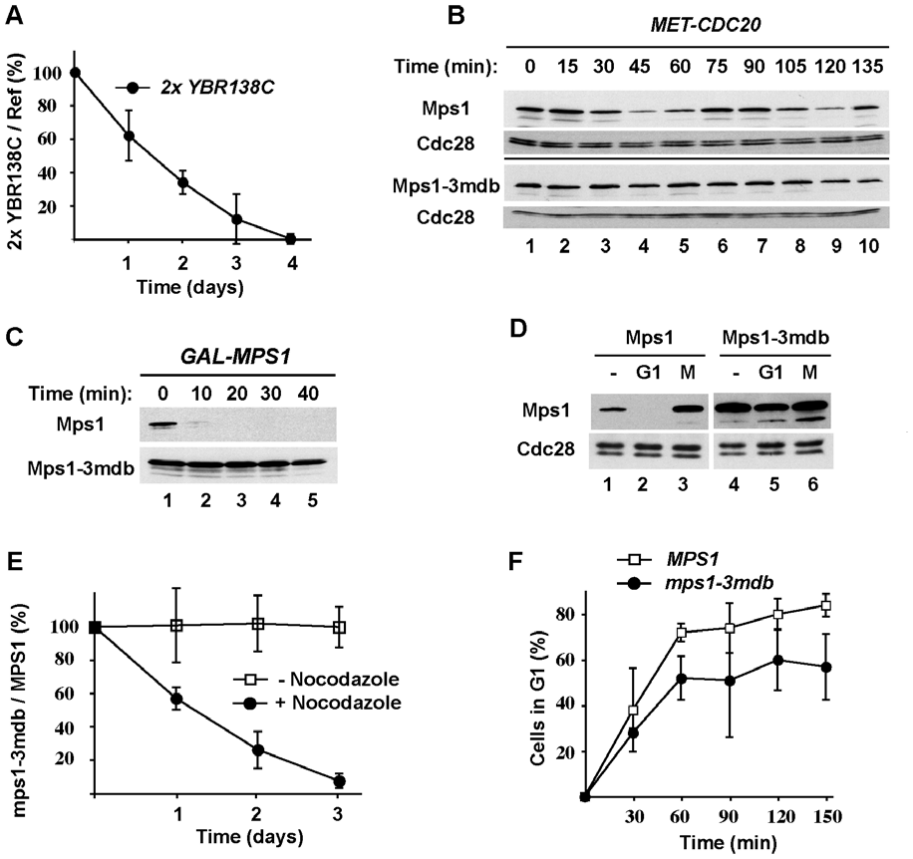
Stabilization of Ybr138C or Mps1 reduces cell fitness. (**A**) Co-cultures of wild-type and *2xYBR138C-TAP* cells. Cells carrying two copies of the *YBR138C* gene were genetically marked (with *TRP1*) and grown together with wild-type (W303) cells (marked with *LEU2*) in complete medium for four days with dilution of the co-cultures once per day. The cultures were tested daily for the presence of *TRP1* and *LEU2* auxotrophs by plating on selective media. The markers were swapped, the experiment repeated, and the averaged results of two trials were plotted. The fraction of *2xYBR138C-TAP* cells on day 0 was arbitrarily set to 100%. (**B**) Cell cycle degradation of Mps1 and Mps1-3mdb. *MET-CDC20* cells carrying endogenously expressed TAP-tagged *MPS1* or *mps1-3mdb* were synchronized in mitosis by incubation with 5 mM methionine for 2.5 hours to deplete Cdc20. Cells were released from the arrest into methionine-free medium. Samples were taken at the indicated times after release and processed for blotting to detect TAP-tagged proteins. (**C**) Stabilization of Mps1 by mutation of its D-boxes. The stabilities of Mps1 and Mps1-3mdb in G1 were assessed as in Figure 2A. (**D**) Elevated expression of Mps1-3mdb. Cells carrying endogenously expressed Mps1 or Mps1-3mdb were arrested in G1 and M phase. The levels of Mps1-TAP were visualized by immunoblotting. Lanes 1 and 4 show the levels of these proteins in cell extracts prepared from asynchronous cell cultures. (**E**) Expression of Mps1-3mdb reduces cell fitness in the presence of mild spindle disruption. *mps1-3mdb* cells were genetically marked and grown together with wild-type *MPS1* cells. Cells were grown in complete medium in the absence (open squares) or presence (closed circles) of 6 µg/ml nocodazole for three days with dilution of the co-cultures once per day. Samples of the cultures were plated daily to determine the relative numbers of *MPS1* and *mps1-3mdb* cells present. The markers were swapped, the experiment repeated, and the averaged results of three independent trials are shown. (**F**) Expression of Mps1-3mdb delays mitotic exit in the presence of mild spindle disruption. Asynchronous cells expressing endogenous *MPS1* (open squares) or *mps1-3mdb* (closed circles) were treated with 100 ng/ml alpha factor in the absence or presence of a semi-permissive concentration of nocodazole (6 µg/ml) at time zero. G1-arrested cells were counted at 30-minute intervals. The averaged results of two trials showing the percentage of cells passing through mitosis into G1 was calculated and plotted. In (A), (E) and (F), error bars denote standard errors.

The data presented in Figures 2 and 3 established that Ybr138C is an APC/C^Cdh1^ substrate; to determine whether Ybr138C degradation is important for cell cycle progression, we compared the growth rates of a wild-type strain and a strain expressing a stabilized form of Ybr138C. However, Ybr138C-ΔN80 was expressed at significantly lower levels than its wild-type counterpart (data not shown). Therefore, instead of testing whether stabilization of Ybr138C affects cell growth, we compared the growth of cells containing one versus two copies of *YBR138C*. We genetically marked the *2xYBR138C* strain and a wild-type strain (W303) so that they could be distinguished on appropriate plates. We then mixed the *2xYBR138C* and wild-type cells and grew them together in co-cultures for four days with large dilutions each day so that even small differences in growth rate could be detected. We found that addition of a second copy of *YBR138C* hindered cell growth; after three days of co-culture, the fraction of *2xYBR138C* cells in the culture was only 12% as much as it was on day zero (Figure 6A). This growth defect amounted to a loss of relative fitness of 13.2% per generation. The most likely explanation for this reduction in fitness is that the *2xYBR138C* cells grow more slowly than wild-type cells, completing just 86.8% of a cell cycle in the time taken by wild-type cells to complete a full cell cycle. In the co-culture experiments, cells slow their growth as the cultures reach densities of OD_600_ of 1–2 prior to daily dilutions. Thus, some of the fitness reduction could also be due to a slower recovery from these higher cell densities. We should point out, however, that these maximal densities are well below stationary phase levels and that the cells never enter a quiescent state. Thus, even a modest increase in Ybr138C level reduces cell fitness, apparently by delaying cell cycle progression. This finding supports the previous observation that *GALp-YBR138C* overexpression was detrimental for cell growth [44]. Conversely, we found that deletion of *YBR138C* had minimal effects on growth in a co-culture experiment, in agreement with previous findings (data not shown; [45]).

### Mps1 stabilization impairs growth in the presence of mild spindle disruption

We next examined whether the degradation of Mps1 is important for proper cell cycle progression. Mps1 is an essential protein kinase with roles in the spindle assembly checkpoint and duplication of the spindle pole body [21]. While this work was in progress, Mps1 was found to be an APC/C^Cdc20^ substrate whose forced expression in anaphase can re-activate the spindle assembly checkpoint [40]. Independently, we found that mutation within a single D-box, ^356^REVL, significantly stabilized Mps1 and that simultaneous mutation of three D-boxes, ^356^REVL, ^267^RELL, ^319^RRAL, completely stabilized Mps1 in G1 cells (Figure 6C). As the only Mps1 in the cell, Mps1-3mdb is fully functional in that it can rescue the essential functions of Mps1 (see below) as well as activation of the spindle checkpoint (not shown). Compared to wild-type Mps1, the levels of endogenous Mps1-3mdb were elevated throughout the cell cycle, particularly during G1 (Figure 6D). In cells synchronized in mitosis and then released, Mps1 levels were high in mitosis, declined approximately 45 minutes after release, and then increased again in the next cell cycle, indicating that endogenous Mps1 is degraded mostly in G1, presumably via APC/C^Cdh1^ (Figure 6B, top panels). In contrast, Mps1-3mdb levels were essentially constant during the cell cycle (Figure 6B, lower panels), confirming its stabilization.

We examined whether Mps1 stabilization affected cell cycle progression. Since Mps1 overexpression activates the spindle checkpoint, leading to a permanent cell cycle arrest [20], we expected that Mps1 stabilization would produce a cell cycle phenotype or delay in cell growth. Surprisingly, we observed no obvious phenotype for *mps1-3mdb* cells under normal growth conditions. To compare the growth rates of *MPS1* and *mps1-3mdb* cells with greater sensitivity, we grew these cells in a single co-culture and determined the fraction of the culture represented by the *mps1-3mdb* cells over time. Under normal growth conditions, the *mps1-3mdb* cells showed no detectable loss of fitness over three days of competitive growth (Figure 6E, open squares).

We performed additional co-culture experiments in the presence of a low, non-lethal dose of the spindle checkpoint-activating drug nocodazole. This treatment causes mild disruption of the mitotic spindle and transient activation of the spindle checkpoint. Under these conditions, we found that the fraction of *mps1-3mdb* cells in the culture declined to just 16% after three days, corresponding to a reduction in fitness of 11.7% (Figure 6E, filled circles). Finally, we directly measured progression of *mps1-3mdb* through mitosis by adding alpha-factor to asynchronous cells and monitoring the accumulation of cells in G1 phase. Relative to cells expressing wild-type Mps1, cells expressing Mps1-3mdb showed delayed progression through mitosis in the presence of a low concentration of nocodazole (Figure 6F). Thus, although Mps1 degradation is not required for cell cycle progression under optimal growth conditions, it becomes important upon mild interference with the mitotic spindle. It is possible that stabilization of Mps1 hyper-sensitizes cells to spindle damage and activation of the spindle checkpoint.

## Discussion

We have developed two complementary approaches for the identification of APC/C substrates. The first approach identified candidates encoded by genes whose cell-cycle expression paralleled that of known APC/C substrates and the second approach used a yeast two-hybrid library to identify proteins that interacted with the APC/C activator Cdh1. Using these approaches we have identified five potential APC/C^Cdh1^ substrates, providing insight into additional cell cycle processes regulated by the APC/C. All of these proteins were unstable in G1 and were either stabilized or partially stabilized by APC/C^Cdh1^ mutations. As additional support, several of these proteins were destabilized following expression of the constitutively active Cdh1-m11 protein and interacted with Cdh1 *in vitro*. Of the proteins we examined, we would classify Fir1, Mps1 and Ybr138C as proven APC/C substrates, Tos4 as a likely substrate, and Pdr3 as a potential substrate requiring further study. With the exception of Mps1, the functions of the proteins identified in this study are relatively unknown. Given that the majority of characterized APC/C substrates function in cell cycle regulation, we anticipate that most of the proteins we identified will also have roles in cell division. We summarize what is known about the functions of these proteins below.

Fir1 has been implicated in mRNA processing. It interacts with RNA cleavage/polyadenylation factors and its deletion results in the shortening of polyA tails [46], [47]. Fir1 interacts with SUMO, a small ubiquitin-like modifier, and contains a SUMO-interacting motif (SIM) [48], [49]. Although we found that Fir1 interacts with Cdh1 and that it is an APC/C^Cdh1^ substrate, mutations within eight putative D-boxes did not stabilize Fir1. Understanding how APC/C-mediated degradation of Fir1 impacts the cell cycle will need to await the identification of a stabilized form of the protein.

We found that Ybr138C expression is cell cycle regulated, that it is an APC/C^Cdh1^ substrate in G1, and that its elevated expression delays cell growth. Together, these findings suggest that the degradation of Ybr138C is important for normal cell cycle progression. Although we were unable to stabilize Ybr138C through mutations within three potential D-boxes, we found that deletion of the N-terminal region, which lacks any obvious degradation motifs, increased protein stability. A similar situation exists with Cik1, a kinesin-associated protein that is expressed as two isoforms with the longer, mitotic form of Cik1 being targeted for ubiquitination by APC/C^Cdh1^ through a unique N-terminal region [50]. Although both Cik1 and Ybr138C contain several D-box motifs, their instability depended upon an unidentified motif(s) within their first 80 amino acids. Interestingly, the interaction of the N-terminal portion of Ybr138C with Cdh1 in the yeast two-hybrid system required Cdh1 residues important for D-box interaction suggesting that this new motif(s) could interact with Cdh1 in a similar manner despite the absence of obvious D-box motifs. More detailed analyses will be required to identify these non-canonical degradation motifs.

Mps1 is a multifunctional protein kinase required for activation and maintenance of the spindle assembly checkpoint (SAC) and for proper duplication of the spindle pole bodies [20], [21]. While this work was in progress, Mps1 was reported to be an APC/C^Cdc20^ substrate whose overexpression in anaphase could reactivate the spindle checkpoint [40]. The Ufd2 ubiquitin ligase has also been implicated in Mps1 degradation [51]. We observed that the bulk of endogenous Mps1 was degraded in G1, though we cannot exclude the possibility that some kinetochore-bound Mps1 might be degraded in mitosis. Given that Mps1 overexpression can activate the spindle checkpoint [20], we were surprised that expression of a stabilized form of Mps1 (Mps1-3mdb) resulted in neither toxicity nor a cell cycle delay, despite its overexpression by several-fold. Interestingly, we found that in the presence of a low concentration of nocodazole, cells expressing Mps1-3mdb delayed progression through mitosis and had reduced cell fitness compared to wild-type cells. Thus, Mps1 degradation is nonessential under normal growth conditions but important for the proper exit from the spindle assembly checkpoint.

Screening of a yeast two-hybrid library for Cdh1-interacting proteins identified two transcription factors, Tos4 and Pdr3, both of which were found to be unstable and whose degradation was at least partially dependent upon APC/C^Cdh1^. For Tos4, we identified four carboxyl-terminal D-boxes and a KEN box that were important for both interaction with Cdh1 and ubiquitination by APC/C^Cdh1^ *in vitro*. We have not yet investigated what motifs are important for Pdr3 degradation. However, it is interesting to note that the Pdr3 protein isolated in the yeast two-hybrid screen consisted of amino acids 411–976, which contains all five putative D-boxes within Pdr3.

At present, we do not know how APC/C^Cdh1^-mediated degradation of either Tos4 or Pdr3 influences cell cycle progression. A recent study demonstrated that Tos4 levels increase dramatically upon DNA replication stress in a Rad53-dependent manner [52]. Interestingly, Pdr3 has also been implicated in the DNA damage response, independent of its role in the pleiotropic drug response pathway. Pdr3, but not the related transcription factor Pdr1, co-activates the transcription of the DNA damage inducible genes *MAG1* and *DDI1* through binding a bi-directional promoter element located between these two genes [43]. In *pdr3Δ* cells, these genes were not induced upon exposure to DNA damaging agents. Since both Tos4 and Pdr3 have been linked to the DNA damage response, it is tempting to speculate that their turnover may be important for resuming cell division after DNA repair. Since we found that APC/C inactivation leads to only partial stabilization of Tos4 and Pdr3, it is likely that other E3s may also regulate their stability, as has been demonstrated for other APC/C substrates [27], [51]. Therefore, considerable effort will be required to design stabilized mutant versions of these proteins in order to investigate how their degradation might influence cell cycle progression after DNA damage.

Various systematic screens for APC^Cdh1^ substrates have been performed and different substrate proteins have been uncovered in each of these screens. Likewise, in the present study we have identified APC/C^Cdh1^ substrates that were not previously found using other systematic approaches. This outcome may have to do with differences in how the screens were conducted. For example our first approach analyzed endogenous low-abundance proteins rather than screening libraries of fluorescently-tagged proteins, which favored identification of more abundant substrates [50]. In addition, we screened a yeast two-hybrid library to identify proteins that specifically interacted with Cdh1 in a D-box dependent manner rather than looking at protein instability as a first step in the screening process. Initial screening for Cdh1 binding may be useful for uncovering proteins that are targeted for ubiquitination by more than one ubiquitin ligase or that are recognized by APC/C^Cdh1^ under different physiological conditions, such as during environmental stresses. For instance, the APC/C targets heat shock factor 2 and the DNA damage checkpoint protein Rad17 in response to protein-damaging and genotoxic stresses, respectively [53], [54]. Similarly, the APC/C may target Tos4 and Pdr3 for degradation following similar stresses and Mps1 for degradation after exposure to the spindle disrupting agent nocodazole.

Even though *CDH1* is non-essential, *cdh1Δ* cells grow slowly and exhibit various morphological abnormalities [22]. Upon superficial analysis, stabilization of none of the Cdh1 substrates identified in this study caused major cell cycle delays. However, overexpression of Mps1, Pdr3, or Tos4 is toxic for cell growth, and overexpressed Fir1 leads to accumulation of sumoylated proteins [48], [49], [52]. Our co-culture experiments revealed that stabilization of Mps1 and elevated expression of Ybr138C reduced cell fitness in the presence and absence of nocodazole, respectively. Similarly, stabilization of two other APC/C^Cdh1^ substrates, Nrm1 and Yhp1, also resulted in modest reductions in cell fitness [27]. The combined effects of stabilizing many such “minor” Cdh1 substrates likely contributes to the stronger phenotype exhibited by *cdh1Δ* cells and to the coordination of cell cycle transitions with other cellular events.
